# Complete genome sequence of *Klebsiella variicola* subsp. *variicola* ML.9ba2, an endophytic strain isolated from aerial roots of *Philodendron erubescens*

**DOI:** 10.1128/mra.01163-23

**Published:** 2024-03-21

**Authors:** M. Lam, K. M. Leung, G. K. K. Lai, F. C. C. Leung, S. D. J. Griffin

**Affiliations:** 1 Shuyuan Molecular Biology Laboratory, The Independent Schools Foundation Academy, Hong Kong, China; University of Strathclyde, Glasgow, United Kingdom

**Keywords:** *Klebsiella variicola*, endophytes, nitrogen fixation, phosphate solubilizing (solubilizing), plant growth promotion

## Abstract

The endophytic strain *Klebsiella variicola* subsp. *variicola* ML.9ba2 was isolated from aerial roots of *Philodendron erubescens* in Hong Kong. Its complete genome of 5,682,083 bp (57.29% G+C), comprising a single chromosome and an IncF plasmid, was established through hybrid assembly.

## ANNOUNCEMENT

While *Klebsiella pneumoniae* species complex (KpSC) members are clinically relevant as opportunistic human pathogens (1) with intrinsic resistance to penicillins (2, 3) often extending to cephalosporins (4, 5) and carbapenems (6, 7), they are also found as endophytes (8). Kingdom-crossing is especially pronounced in strains of *K. variicola* (9), which are not only nitrogen fixers and plant colonizers, isolated from sources such as sugarcane (10), rice and bananas (11), but also found in bloodstream infections (12), cattle urine (13) and termite gut microbiota (14).

Here, ML.9ba2 was isolated from aerial roots of *Philodendron erubescens* in Hong Kong (22.266384N, 114.191128E; 2021-06-25). 20 mm lengths of three root tips from a single plant were surface-sterilized using 1% wt:vol 8-hydroxyquinoline sulfate before incubation in nitrogen-free Jensen’s broth M710 (15, 16) for 7 days at 32°C. Following subculture to fresh broth and incubation under the same conditions, a 100 µL aliquot was spread on M710 agar and the resultant colonies tested for phosphate solubilization on Pikovskaya’s agar (17). A single colony of ML.9ba2, which grows well on M710 and gives clear zones on Pikovskaya’s agar, was passaged to purity on M710 agar before streaking a single colony onto Luria agar (18) and harvesting for DNA extraction following overnight growth (DNeasy PowerSoil Pro kit, Qiagen GmbH, Hilden, Germany). All agar incubations conducted at 27°C.

Paired-end short-read sequencing libraries were prepared using a NexteraXT DNA Library Preparation Kit (Illumina, Inc., USA) and sequenced *via* the Illumina MiSeq platform using v3 chemistry (2 × 300 bp). 798,646 raw read pairs were quality-filtered and trimmed using TrimGalore! v0.6.7 (https://github.com/FelixKrueger/TrimGalore) (stringency:3; -e:0.2), producing 790,949 read pairs (median length 170 bp) totaling ~279 Mbp (SRX21353898). Long-read libraries, prepared from the same extracted DNA using the Rapid Barcoding Kit SQK-RBK004, were sequenced using a Spot-ON Flow Cell (vR9) and MinION sequencer, with MinKNOW v3.1.8 software and base-calling by Guppy v2.1.3 high-accuracy mode (all from Oxford Nanopore Technologies plc, UK). The final long-read data set of 429,202 reads was trimmed by Filtlong v0.2.1 (https://github.com/rrwick/Filtlong) (min_length:2000; target_bases:350Mbp; length_weight:10) to give 56,702 reads (350 Mbp) (SRX21353899) with a median length of 4,712 bp (N50 7,681). Default parameters were used for all software unless otherwise specified.

Hybrid assembly of MiSeq and MinION filtered data sets, overlap removal, and sequence rotation were performed using Unicycler v0.4.3 (19) to yield a circular chromosome of 5,515,756 bp (57.48% G+C) and a circular plasmid (166,327 bp, 51.1% G+C), rotated to begin DNAA_KLEP3 (*dnaA*) and Q6U5J6_KLEPN (*repA*), respectively. The genome was judged 100% complete (0.36% contamination) by CheckM v.1.1.6 (20) and submitted to NCBI PGAP v6.4 (21) for annotation. Mash/MinHash at BV-BRC 3.34.11 (22) found ML.9ba2 closest to *Klebsiella variicola* subsp. *variicola* strain F2R9T (CP084765) with an average nucleotide identity of 99.09% (23).

PGAP predicted numerous genes associated with plant-growth promotion and endophytism (Table 1) (24–28) as well as a chromosomally encoded LEN-19 beta-lactamase (MKK01_RS13570) (29). Plasmid pML9ba2 was classified as type IncFIB(K)_5(cladeI) by the KpVR web-based tool (https://bioinfo-mml.sjtu.edu.cn/KpVR/index.php), which found no antimicrobial resistance genes.

**TABLE 1:** Predicted genes^*a*^ in ML.9ba2 potentially linked to plant-growth promotion and endophytism

Gene(s)	Protein(s)	Locus	Function
*nif* cluster	*nifJHDKTXENXUSVWZMFLABQ*	MKK01_RS08305 to MKK01_RS08400	Nitrogen fixation
*Pho* regulon	*phoPQ*, *phoH*, *phoBR*, *pstSCAB-phoU*	MKK01_RS16770 to MKK01_RS16775, MKK01_RS17230,	Phosphate solubilization and utilization
		MKK01_RS20955 to MKK01_RS20950,	
		MKK01_RS26375 to MKK01_RS26395,	
*aphA*	Acid phosphatase	MKK01_RS24820	
*phoA*	Alkaline phosphatase	MKK01_RS21020	
*phy^b^*	Phytase^*b*^	MKK01_RS19375	
*bcs* operon	*bcsOQABCDZ* (type Ib), *bcsGFERQABZC* (type IIa),	MKK01_RS01075 to MKK01_RS01150	Cell-wall modification
*chiA^b^*	Chitinase (EC 3.2.1.14)^*b*^	MKK01_RS15875	Fungal defense
*nhhABG*	Nitrile hydratase (EC 4.2.1.84)	MKK01_RS13035 to MKK01_RS13045	Indole-3-acetic acid (IAA) synthesis, auxin transport, and plant ontogenesis.
*ami*	Amidase (EC 3.5.1.4)	MKK01_RS13030	
*tynA*	Monoamine oxidase (EC 1.4.3.4)	MKK01_RS14785	
*aldB^b^*	Aldehyde dehydrogenase(EC 1.2.1.3)^*b*^	MKK01_RS05580, MKK01_RS19890	
*aec*	Auxin efflux carrier (AEC) family transporter	MKK01_RS05085, MKK01_RS09265, MKK01_RS14270	
*ent-fep* gene cluster	*entHABEC-fepB-entS-fepDGC-* *entF-mbtH-fes-fepA-entD*	MKK01_RS19440 to MKK01_RS19510	Enterobactin biosynthesis and transport, root colonization through ROS defense
*cirA, fiu*	Ferric catecholate siderophore receptor	MKK01_RS07705, MKK01_RS15625	Iron-catecholate transport, plant colonization
*fhu* operon	*fhuABCD, fhuE, fhuF,* ferric hydroxamate transport operon	MKK01_RS21875 to MKK01_RS21890, MKK01_RS22900	Ferrichrome transport and utilization, albomycin uptake

^*a*^ Gene assignments by NCBI PGAP v6.4.

^*b*^ Further identification by homology *via* UniProt (30).

## Data Availability

The complete genome sequence and unfiltered sequence data for *Klebsiella variicola* subsp. variicola ML.9ba2 are available through NCBI under BioProject PRJNA809577, with GenBank and SRA accession numbers CP092632 (chromosome), CP092633 (plasmid), SRX22552494 (MinION), and SRX22631600 (MiSeq).
